# Dynamics Matter: Recognition of Reward, Affiliative, and Dominance Smiles From Dynamic vs. Static Displays

**DOI:** 10.3389/fpsyg.2018.00938

**Published:** 2018-06-11

**Authors:** Anna B. Orlowska, Eva G. Krumhuber, Magdalena Rychlowska, Piotr Szarota

**Affiliations:** ^1^Institute of Psychology, Polish Academy of Sciences, Warsaw, Poland; ^2^Department of Experimental Psychology, University College London, London, United Kingdom; ^3^School of Psychology, Queen’s University Belfast, Belfast, United Kingdom

**Keywords:** smile, facial expression, emotion, dynamic, mimicry

## Abstract

Smiles are distinct and easily recognizable facial expressions, yet they markedly differ in their meanings. According to a recent theoretical account, smiles can be classified based on three fundamental social functions which they serve: expressing positive affect and rewarding self and others (reward smile), creating and maintaining social bonds (affiliative smile), and negotiating social status (dominance smiles) (Niedenthal et al., 2010; Martin et al., 2017). While there is evidence for distinct morphological features of these smiles, their categorization only starts to be investigated in human faces. Moreover, the factors influencing this process – such as facial mimicry or display mode – remain yet unknown. In the present study, we examine the recognition of reward, affiliative, and dominance smiles in static and dynamic portrayals, and explore how interfering with facial mimicry affects such classification. Participants (*N* = 190) were presented with either static or dynamic displays of the three smile types, whilst their ability to mimic was free or restricted via a pen-in-mouth procedure. For each stimulus they rated the extent to which the expression represents a reward, an affiliative, or a dominance smile. Higher than chance accuracy rates revealed that participants were generally able to differentiate between the three smile types. In line with our predictions, recognition performance was lower in the static than dynamic condition, but this difference was only significant for affiliative smiles. No significant effects of facial muscle restriction were observed, suggesting that the ability to mimic might not be necessary for the distinction between the three functional smiles. Together, our findings support previous evidence on reward, affiliative, and dominance smiles by documenting their perceptual distinctiveness. They also replicate extant observations on the dynamic advantage in expression perception and suggest that this effect may be especially pronounced in the case of ambiguous facial expressions, such as affiliative smiles.

## Introduction

A smile can be simply described as a contraction of the *zygomaticus major* - a facial muscle which pulls the lip corners up toward the cheekbones (Ekman and Friesen, 1982), named by Duchenne de Boulogne (1862/1990) “a muscle of joy.” This unique movement makes it an easily recognizable facial expression. However, smiles can also be confusing in their meanings and functions they serve. Despite the association between smiles and positive feelings and intentions (Ekman et al., 1990), trust (Krumhuber et al., 2007) and readiness to help (Vrugt and Vet, 2009), smiles can also be displayed during unpleasant experiences, i.e., to hide negative feelings (Ekman and Friesen, 1982), and be perceived as a signal of lower social status (Ruben et al., 2015) or intelligence (Krys et al., 2014). Smiling is therefore used in a wide variety of situations, depending on the context and social norms learned through socialization and experience. Not only can the use of smiles and their social function vary considerably (e.g., Szarota et al., 2010; Rychlowska et al., 2015), but the very expression of a smile comes in many forms. This is because the contraction of the *zygomaticus major* muscle [defined as Action Unit (AU) 12 in the Facial Action Coding System; Ekman et al., 2002] – the core feature of any smile expression – often involves the activation of other facial muscles, creating a range of possible variations. Ekman (2009), for example, identified and described 18 types of smiles, differentiated in terms of their appearance and the situation in which they are likely to occur. Moreover, AU12 can be accompanied by the presence of other AUs and thus convey emotions such as disgust or surprise (Du et al., 2014; Calvo et al., 2018).

Despite its variability, the most commonly used smile typology is the distinction between ‘true’/genuine and ‘fake’/false smiles, with the former being sincere displays of joy and amusement, and the latter being produced voluntarily, possibly to increase others’ trust and cooperation (Frank, 1988). True and false smiles can be distinguished on the basis of their morphology: the presence of supposedly involuntary eye constriction (AU6 – the contraction of the *orbicularis oculi* muscle), a classic criterion based on early studies by Duchenne de Boulogne (1862/1990). Although the true vs. false smile typology is parsimonious and extensively documented in the literature, it is not without shortcomings. Specifically, contemporary empirical evidence reveals that people are able to deliberately show Duchenne smiles (Krumhuber and Manstead, 2009; Gunnery et al., 2013), thereby limiting the usefulness of this criterion. More importantly, however, the binary nature of the typology fails to account for the variability of smiles produced in everyday life. People smile in many situations, involving diverse emotions or very little emotion. Some expressions undeniably convey more positive affect than others. However, the assertion that all smiles which fail to reflect joy and amusement must be false and potentially manipulative, seems oversimplifying. It is at least theoretically possible that an enjoyment smile is just one among many true smiles.

An alternative theoretical account proposes that smiles can be classified in accordance to how they affect people’s behavior in the service of fundamental tasks of social living (Niedenthal et al., 2010; Martin et al., 2017). This typology defines three physically distinct smiles of *reward*, *affiliation*, and *dominance*, which serve the main function of social communication and interaction (Niedenthal et al., 2013). Reward smiles communicate positive emotions and sensory states such as happiness or amusement, thereby potentially rewarding both the sender and the perceiver. Affiliative smiles communicate positive social motives and are used to create and maintain social bonds. A person displaying an affiliative smile intends to be perceived as friendly and polite. Finally, dominance smiles are used to impose and maintain higher social status. The person displaying this type of smile intends to be perceived as superior. Recent research by Rychlowska et al. (2017, Study 1) explored the physical appearance of reward, affiliative, and dominance smiles, including a description of the facial characteristics of each category, suggesting that the three functional smiles are indeed morphologically different. In a subsequent experiment (Rychlowska et al., 2017, Study 2), computer-generated animations of reward, affiliative, and dominance smiles were categorized by human observers and a Bayesian classifier. Despite the generally high categorization accuracy for all three smile types, human and Bayesian performance was lowest for the affiliative smiles, arguably because of their similarity to the reward smiles, as both expressions convey positive social signals and they both involve a symmetrical movement of the *zygomaticus major* muscle.

Given the multiple types of smiles, the diversity of situations in which they appear, and the varying display rules governing their production, the understanding of these facial expressions is a complex process which can rely on multiple mechanisms – such as a perceptual analysis of the expresser’s face, conceptual knowledge about the expresser and the situation, and sensorimotor simulation (Niedenthal et al., 2010; Calvo and Nummenmaa, 2016). This last construct involves the recreation of smile-related feelings and neural processes in the perceiver, and is closely related to facial mimicry, which is defined as a spontaneous rapid imitation of other people’s expressions (Dimberg et al., 2000). As sensorimotor simulation involves a complex sequence of motor, neural, and affective processes (see Wood et al., 2016, for review), it is more costly than other forms of facial expression processing. Hence, it may be preferentially used for the interpretation of expressions that are important for the observer or non-prototypical, and thus hard to classify (Niedenthal et al., 2010).

Existing literature suggests that facial mimicry, often used to index sensorimotor simulation of emotion expressions, is sensitive to social and contextual factors. Its occurrence may depend on the type of expression observed (Hess and Fischer, 2014), but also on the social motivation (Fischer and Hess, 2017), attitudes toward the expresser (e.g., Likowski et al., 2008), and group status (e.g., Sachisthal et al., 2016). Furthermore, it can be experimentally altered or restricted in laboratory settings using various pen-in-mouth procedures, stickers, chewing-gum, or sports mouthguards. In these cases, preventing mimicry responses has been shown to impair observers’ ability to accurately recognize happiness and disgust (Oberman et al., 2007; Ponari et al., 2012) and discriminate between false and genuine smiles (Maringer et al., 2011; Rychlowska et al., 2014).

Parallel to these findings, the results of other studies investigating the role of facial mimicry in emotion recognition were not conclusive (e.g., Blairy et al., 1999; Korb et al., 2014). Several factors could explain such inconsistencies: First, measuring rather than blocking facial mimicry may not necessarily show its involvement in expression recognition. Also, facial mimicry could be more implicated in recognition tasks that are especially difficult, i.e., when classifying low-intensity facial expressions or judging subtle variations between different types of a given facial expression (Hess and Fischer, 2014). This makes the interpretation of smiles an especially useful paradigm for studying the role of facial mimicry.

Another potential explanation for disparate research findings could be related to the way in which the stimuli are presented. Previous studies using facial electromyography (EMG; e.g., Sato et al., 2008; Rymarczyk et al., 2016) reveal that dynamic video stimuli lead to enhanced mimicry in comparison with static images. In particular, higher intensities of AU12 and AU6 – the core smile movements – have been reported when participants watched dynamic rather than static expressions of happiness. Dynamic materials have higher ecological validity (Krumhuber et al., 2013, 2017), given that in everyday social encounters facial expressions are moving and rapidly changing depending on the situation. As emotion processing is not only based on the perception of static configurations of facial muscles, but also on how the facial expression unfolds (Krumhuber and Scherer, 2016), dynamic displays provide additional information which is not present in static images. Furthermore, past research reveals better recognition and higher arousal ratings of emotions when they are shown in dynamic than static form (e.g., Hyniewska and Sato, 2015; Calvo et al., 2016). Dynamic displays may therefore provide relevant cues which facilitate the decoding of facial expressions.

The present work focuses on the distinction between the three functional smiles of reward, affiliation, and dominance (Niedenthal et al., 2010). Instead of using computer-generated faces as done by Rychlowska et al. (2017), we employed static images and dynamic videos of human actors displaying the three types of smiles. Our experiment extends previous research (Rychlowska et al., 2017; Martin et al., 2018) in three ways by testing (1) how accurately naïve observers can discriminate between the three functional smiles, (2) whether the capacity to classify these smiles is affected by facial muscle restriction that prevents mimicry responses, and (3) whether the type of display (static vs. dynamic) influences smile recognition, thereby moderating the potential effects of muscle restriction. In line with previous findings (Rychlowska et al., 2017), we predict that observers should be able to accurately classify the three functional smiles, with affiliative smiles being more ambiguous than reward and dominance smiles. We also anticipate that, consistent with previous work (Maringer et al., 2011; Rychlowska et al., 2014), facial muscle restriction should disrupt participants’ ability to interpret the three smile types. Finally, we hypothesize that impairments in smile classification in the muscle restriction condition should be especially strong in the static, rather than dynamic condition, given the relative smaller amount of information provided by stimuli of static nature.

## Materials and Methods

### Participants and Design

The study had a three-factorial experimental design with the stimulus display (dynamic vs. static) and muscle condition (free vs. restricted) as between-subject variables, and smile type (reward, affiliative, dominance) as within-subject variable. A total of 190 participants, mostly students at University College London, were recruited and voluntarily took part in the study in exchange for a £2 voucher or course credits. One hundred seventy-eight subjects identified themselves as White and 12 as mixed race. Technical failure resulted in the loss of data for two participants, leaving a final sample of 188 participants (137 women), ranging in age between 18 and 45 years (*M* = 22.2 years, *SD* = 4.2). A power analysis using G^∗^Power 3.1 (Faul et al., 2007) for a 3 × 2 × 2 interaction, assuming a medium-sized effect (Cohen’s *f* = 0.25) and a 0.5 correlation between measures, indicated that this sample size would be sufficient for 95% power. All participants had normal or corrected-to-normal vision. Ethical approval for the present study was granted by the UCL Department of Psychology Ethics Committee.

### Materials

**FIGURE 1:**
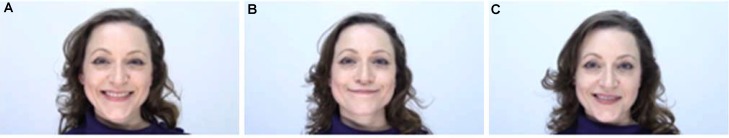
Example of a reward smile **(A)**, affiliative smile **(B)**, and dominance smile **(C)** at the peak intensity of the display.

Stimuli were retrieved from a set developed by Martin et al. (2018) and featured eight White actors (four female) in frontal view, expressing the three smile types: reward smile (eight stimuli), affiliative smile (eight stimuli), and dominance (six stimuli) smile. Actors posed each smile type after being coached about its form and accompanying social motivations (see Martin et al., 2017; Rychlowska et al., 2017). In morphological terms (FACS, Ekman et al., 2002), reward smiles consisted of Duchenne smiles that were characterized by symmetrical activation of the Lip Corner Puller (AU12), the Cheek Raiser (AU6), Lips Part (AU25) and/or Jaw Drop (AU26). Affiliative smiles consisted of Non-Duchenne smiles that involved the Lip Corner Puller (AU12), the Chin Raiser (AU17), with or without Brow Raiser (AU1-2). Dominance smiles consisted of asymmetrical Non-Duchenne smiles (AU12L or AU12R), with additional actions, such as Head Up (AU53), Upper Lip Raiser (AU10), and/or and Lips Part (AU25) (see **Figure 1**). We employed both static and dynamic portrayals of each smile expression, netting 22 static and 22 dynamic stimuli. Dynamic stimuli were short videoclips (2.6 s) which showed the face changing from non-expressive to peak emotional display. Static stimuli consisted of a single frame of the peak expression. All stimuli were displayed in color on white backgrounds (size: 960 × 540 pixels).

### Procedure

Participants were tested individually in the laboratory. After providing informed consent, they were randomly assigned to one of the four experimental conditions, resulting in approximately 47 people per cell. Using the Qualtrics software (Provo, UT, United States), participants were instructed that they would view a series of smile expressions. Their task was to classify the smiles into three categories. The following brief definitions of each smile type, informed by previous research Rychlowska et al. (2017), were provided: (a) *reward smile*: “a smile displayed when someone is happy, content or amused by something,” (b) *affiliative smile*: “a smile which communicates positive intentions, expresses a positive attitude to another person or is used when someone wants to be polite,” and (c) *dominance smile*: “a smile displayed when someone feels superior, better and more competent or wants to communicate condescension toward another person.”

In addition to these smile descriptions, participants were given examples of situations in which each type of expression was likely to occur: (a) *reward smile*: “being offered a dream job or seeing a best friend, not seen for a long time,” (b) *affiliative smile:* “entering a room for a job interview or greeting a teacher,” (c) *dominance smile:* “bragging to a rival about a great job offer, meeting an enemy after winning an important prize.” Situational descriptions were pre-tested in a pilot study, in which participants (*N* = 33) were asked to choose amongst the three functional smiles the expression that best matched a particular situation (from a pool of 13 situational descriptions). For the present study, we selected the situation that was judged to be the most appropriate for each type of smile expression (selection frequency: reward: 94%, affiliative, 93%, dominance: 75%).

During the muscle restriction condition, participants were informed that people were more objective in their judgments of emotions when their facial movements were restrained. A similar cover story has been used by Maringer et al. (2011). In order to inhibit the relevant facial muscles, participants were to hold a pencil sideways, using both lips and teeth, without exerting any pressure (for a similar procedure see Niedenthal et al., 2001; Maringer et al., 2011). The experimenter demonstrated the correct way of holding the pen in the mouth, and only after the experimenter was satisfied with the pen holding technique, the experiment was started. There was no additional instruction in the free muscle condition.

After some comprehension checks of the three types of smile expressions, participants were presented with static or dynamic versions of the 22 stimuli, shown in a random sequence at the center of the screen. Dynamic sequences were played in their entire length; static photographs were displayed for the same length as the videos (2.6 s). For each stimulus, participants rated their confidence (from 0 to 100%) about the extent to which the expression was a reward, an affiliative, or a dominance smile. If they felt that more than one category applied, they could respond using multiple sliders to choose the exact confidence levels for each response category. Ratings across the three response categories had to sum up to 100%. We defined classification accuracy as the likelihood of correctly classifying a smile expression in line with the predicted target label (reward, affiliation, dominance). After completion of the experiment, participants were debriefed and thanked.

## Results

### Smile Classification

To test whether the three functional smiles are correctly classified by naïve observers, we calculated the mean confidence ratings for correct (i.e., function-consistent) answers for each smile type (accuracy rates). A 2 (stimulus display: static, dynamic) × 2 (muscle condition: free, restricted) × 3 (smile type: reward, affiliative, dominance) ANOVA, with smile type as within-subjects variable, and classification accuracy as the dependent measure yielded significant main effects of smile type, *F*(2,368) = 17.41, *p* < 0.001, $\eta_{p}^{2}$ = 0.09, and stimulus display, *F*(1,184) = 13.51, *p* < 0.001, $\eta_{p}^{2}$ = 0.07. The two main effects were qualified by a significant interaction between smile type and display, *F*(2,368) = 3.99, *p* = 0.021, $\eta_{p}^{2}$ = 0.02. The main effect of muscle condition, *F*(1,184) = 0.89, *p* = 0.348, $\eta_{p}^{2}$ = 0.01, the smile type by muscle condition interaction *F*(2,368) = 2.71, *p* = 0.070, $\eta_{p}^{2}$ = 0.01, the display by muscle condition interaction *F*(1,184) = 1.90, *p* = 0.170, $\eta_{p}^{2}$ = 0.01, and the smile type, display and muscle condition interaction *F*(2,368) = 0.16, *p* = 0.845, $\eta_{p}^{2}$ = 0.001, were not significant.

**FIGURE 2:**
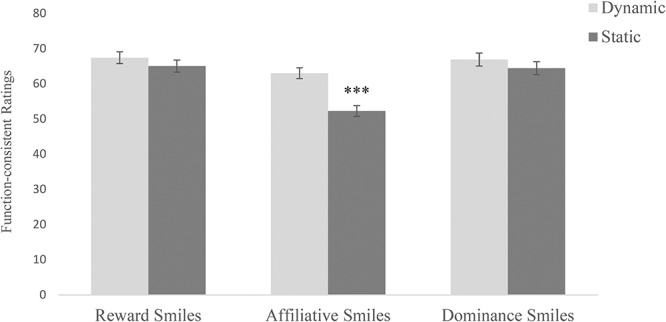
Function-consistent mean ratings (accuracy rates) of the three smile types in the dynamic and static condition. Error bars represent standard errors of the mean. The asterisks indicate a significant difference in the mean ratings between static and dynamic condition (*p* < 0.001).

The main effect of smile type revealed that reward smiles (*M* = 66.25, *SD* = 16.37) and dominance smiles (*M* = 64.47, *SD* = 17.98) were recognized more accurately than affiliative smiles (*M* = 57.70, *SD* = 15.75, *ps* < 0.001, Bonferroni-corrected). The difference in recognition rates between reward and dominance smiles was not significant (*p* = 0.29, Bonferroni-corrected). The main effect of stimulus display revealed that recognition rates of the three smile types were higher in the dynamic (*M* = 65.80, *SD* = 9.92) than static condition (*M* = 59.98, *SD* = 11.71). However, decomposing the significant interaction between smile type and display with simple effects analyses revealed that affiliative smiles were recognized more accurately in the dynamic (*M* = 63.06, *SD* = 13.04) than static condition (*M* = 52.30, *SD* = 16.47), *F*(1,184) = 24.32, *p* < 0.001, $\eta_{p}^{2}$ = 0.12. No significant differences between the dynamic and static condition emerged for the recognition of reward smiles, *F*(1,184) = 0.94, *p* = 0.335, $\eta_{p}^{2}$ = 0.01, and dominance smiles, *F*(1,184) = 2.87, *p* = 0.092, $\eta_{p}^{2}$ = 0.02 (see **Figure 2**).

### Smile Confusions

The confusion matrix in **Table 1** provides a detailed overview of true (false) positives and true (false) negatives in smile classification. In order to analyze the type of confusions within a smile type, we followed established procedures (see Calvo and Lundqvist, 2008) and submitted function-consistent and function-inconsistent ratings of the smile expressions to a 2 (stimulus display: static, dynamic) × 2 (muscle condition: free, restricted) × 3 (smile type: reward, affiliative, dominance) × 3 (response: reward, affiliative, dominance) ANOVA, with smile type and response as within-subjects factors. The results revealed a significant main effect of smile type, *F*(2,368) = 867.54, *p* < 0.001, $\eta_{p}^{2}$ = 0.83, and response, *F*(2,368) = 36.11, *p* < 0.001, $\eta_{p}^{2}$ = 0.16, as well as a significant interaction between the two factors, *F*(4,736) = 726.55, *p* < 0.001, $\eta_{p}^{2}$ = 0.80. The interaction between smile type, response, and stimulus display was also significant *F*(4,736) = 9.20, *p* < 0.001, $\eta_{p}^{2}$ = 0.05. The response by stimulus display interaction, *F*(2,368) = 1.76, *p* = 0.177, $\eta_{p}^{2}$ = 0.01, the smile type, response, and muscle condition interaction *F*(4,736) = 2.28, *p* = 0.080, $\eta_{p}^{2}$ = 0.01, as well as the interaction between smile type, response, stimulus display and muscle condition *F*(4,736) = 1.37, *p* = 0.252, $\eta_{p}^{2}$ = 0.01, were not significant.

**Table 1:** Smile type confusions in the static and dynamic condition.

	Display	Reward ratings	Affiliative ratings	Dominance ratings
Reward smiles	Dynamic	67.44	26.37	6.18
	Static	65.04	27.98	6.97
Affiliative Smiles	Dynamic	9.23	63.03^∗∗∗^	27.75^∗∗∗^
	Static	10.44	52.27^∗∗∗^	37.29^∗∗∗^
Dominance Smiles	Dynamic	9.16	23.93	66.91
	Static	10.58	27.44	61.98

^∗∗∗^*p < 0.001, significant difference in the mean ratings between static and dynamic display*.

To decompose the three-way interaction, we examined the interactive effect of response and display separately for each smile type. The interaction of response (reward, affiliative, dominance) and display (static, dynamic) was not significant for the confusions of reward smiles, *F*(2,372) = 0.78, *p* = 0.459, $\eta_{p}^{2}$ = 0.004, and dominance smiles, *F*(2,372) = 2.8 *p* = 0.062, $\eta_{p}^{2}$ = 0.02, suggesting that the classification of these smiles was similar in both display conditions.

However, the interaction of response and display was significant for the confusion of affiliative smiles, *F*(2,372) = 18.57, *p* < 0.001, $\eta_{p}^{2}$ = 0.09. Overall, these smiles were rated higher on affiliation (*M* = 57.70, *SD* = 15.75) than dominance (*M* = 32.47, *SD* = 15.39) and reward (*M* = 9.83, *SD* = 9.58, *ps* < 0.001), but they were also more likely to be confused with dominance than reward smiles, *F*(2,372) = 408.03, *p* < 0.001, $\eta_{p}^{2}$ = 0.69. Simple effects analyses revealed that affiliative smiles were equally likely to be classified as reward smiles in both display conditions (static: *M* = 10.44, *SD* = 10.88, dynamic: *M* = 9.23, *SD* = 8.13, *p* = 0.386). However, affiliative smiles were also less likely to be accurately classified as affiliative in the static (*M* = 52.27, *SD* = 16.50) than in the dynamic condition (*M* = 63.03, *SD* = 52.27, *p* < 0.001). This difference results from participants rating affiliative smiles as more dominant in the static condition (*M* = 37.29, *SD* = 15.70) than in the dynamic condition (*M* = 27.75, *SD* = 13.58, *p* < 0.001) (see **Table 1**).

## Discussion

The purpose of the present work was to test the extent to which the functional smiles of reward, affiliation, and dominance are distinct and recognizable facial expressions. We also aimed to explore the role of facial muscle restriction and presentation mode in moderating smile classification rates. The results reveal that participants were able to accurately categorize reward, affiliative and dominance smiles. This supports the assumption that diverse morphological characteristics of smiles are identified in terms of their social communicative functions (Niedenthal et al., 2010; Martin et al., 2017). The use of naturalistic human face stimuli, rather than computer-generated faces, extends existing work (Rychlowska et al., 2017), thereby achieving greater ecological validity.

Our results reveal that classification accuracy was significantly lower for affiliative smiles than reward and dominance smiles. This is in line with previous findings by Rychlowska et al. (2017) who showed that human observers and a Bayesian classifier were less accurate in categorizing affiliative smiles compared to reward and dominance smiles (using a binary yes/no classification approach to indicate whether a given expression was – or was not – an instance of a given smile type). The present research used continuous confidence ratings that were not mutually exclusive, thus replicating their findings with human-realistic stimuli and a different response format. Moreover, a closer inspection of participants’ ratings reveals that, whereas affiliative smiles were relatively unlikely to be classified as reward, reward smiles were often judged as affiliative, consistently with the results of Rychlowska et al. (2017) and Martin et al. (2018). While this finding suggests that reward smiles – similarly to the Duchenne smiles previously described in the literature – may constitute a more homogeneous, less variable category than other smiles (e.g., Frank et al., 1993), it also highlights similarities between reward and affiliative smiles which both convey positive social motivations. It is worth noting that participants in the present study saw smile expressions of White/Caucasian targets without any background information. The only context given in the study was the definition of the three smile types including examples of situations in which they might potentially occur. Recent work by Martin et al. (2018) suggests that the three types of smiles elicit distinct physiological responses when presented in a social-evaluative context. Adding social context to these displays therefore provides a promising avenue for future research, as the salience of specific interpersonal tasks could facilitate the distinction between affiliative smiles and the other two categories.

As predicted, the current study revealed higher recognition rates of the expressions presented in dynamic compared to static mode, and this applied in particular to affiliative smiles. This finding corroborates existing research on the dynamic advantage in emotion recognition (Hyniewska and Sato, 2015; Calvo et al., 2016). The fact that presentation mode is particularly important in the recognition of affiliative smiles confirms the assumption that dynamic features might be especially helpful in the identification of more subtle and ambiguous facial expressions, i.e., non-enjoyment smiles (Krumhuber and Manstead, 2009). As such, fundamental differences in the timing of smiles such as amplitude, total duration, and speed of onset, apex, and offset (Cohn and Schmidt, 2004) might inform expression classification (Krumhuber and Kappas, 2005).

Contrary to our predictions and to previous findings (Niedenthal et al., 2010; Maringer et al., 2011; Rychlowska et al., 2014), our results did not support the moderating role of people’s ability to mimic in smile classification. According to Calvo and Nummenmaa (2016), facial expressions consist of morphological changes in the face and their underlying affective content. Given that participants were instructed to rate each smile on three pre-designed scales (reward, affiliative, dominance smile), it is possible that this procedure induced cognitive, label-driven, rather than affective processing based on embodied simulation. Alternatively, the provision of a clear definition of the three functional smiles might have failed to encourage the social motivation necessary for facial mimicry to occur (Hofman et al., 2012; Hess and Fischer, 2014). It is also possible that other factors, i.e., trait empathy (Kosonogov et al., 2015) or endocrine levels (Kraaijenvanger et al., 2017) impact smile recognition rates as well as modulate the occurrence of mimicry. We think that it is unlikely that the present results are caused by an improper technique for blocking mimicry given that the experimenter closely monitored whether participants held the pencils correctly. In addition, we used a reliable facial muscle restriction technique employed in previous studies which revealed the moderating role of mimicry in emotion perception (Niedenthal et al., 2001; Maringer et al., 2011).

One potential limitation of our study was that we did not measure mimicry during the smile classification task. It is thus impossible to conclude whether participants in the free mimicry condition were really mimicking the smiles or whether mimicry occurred but did not enhance recognition performance in comparison to the restricted condition. We therefore suggest for future research on mimicry blocking to use EMG measurements in order to assess the presence of facial mimicry in the free muscle condition as well as the effectiveness of mimicry blocking in the restricted muscle condition. Finally, the lack of significant effects of the muscle restriction procedure may also reflect the complexity of sensorimotor simulation; a process which does not always involve measurable facial mimicry. Given that generating a motor output is a critical component for sensorimotor simulation more than facial activity *per se* (e.g., Korb et al., 2015; Wood et al., 2016), future studies could investigate the extent to which judgments of functional smiles are impaired by experimental manipulations that involve the production of conflicting facial movements.

In sum, the present research investigated observers’ judgments of reward, affiliative, and dominance smiles. While participants were able to accurately categorize each smile type, recognition accuracy was lower for affiliative than for reward and dominance smiles. Although preventing mimicry responses did not appear to influence participants’ classification, the use of dynamic versus static stimuli increased recognition accuracy of affiliative smiles. To our knowledge, this is the first study to test the role of muscle restriction and presentation mode in the recognition of reward, affiliative, and dominance smiles. The results highlight the importance of dynamic information, being particularly salient in the recognition of affiliative smiles which are the most ambiguous among the three smile types. The lack of a significant effect of facial muscle condition on smile classification suggests that the functional smiles can be recognized based on their physical appearance. Our findings contribute to the understanding of the importance of temporal dynamics in the perception of emotional expressions.

## Author Contributions

AO, EK, PS, and MR conceived and designed the experiments. AO performed the experiments. AO and EK performed the statistical analysis. AO wrote the first draft of the manuscript. EK, MR, and PS wrote sections of the manuscript.

## Conflict of Interest Statement

The authors declare that the research was conducted in the absence of any commercial or financial relationships that could be construed as a potential conflict of interest.
